# Negative Interference in Serum HBsAg ELISA from Rheumatoid Factors

**DOI:** 10.1371/journal.pone.0080620

**Published:** 2013-11-15

**Authors:** Lei Xu, Zhen Yu, Wen Fan, Xueping Wang, Mingshui Xie, Yiting Xu, Lihua Hu, Yirong Li

**Affiliations:** 1 Department of Laboratory Medicine , Union Hospital, Tongji Medical College, Huazhong University of Science and Technology, Wuhan, China; 2 Department of Laboratory Medicine, Tongji Medical College, Huazhong University of Science and Technology, Wuhan, China; 3 Department of Laboratory Medicine, Jingzhou First People’s Hospital, Jingzhou, China; 4 Department of Laboratory Medicine, Suizhou central Hospital, Suizhou, China; CRCL-INSERM, France

## Abstract

**Background:**

RF(Rheumatoid factor) is usually thought to cause positive interference in immunoassay. Recently, our study showed that high-concentration RFs caused negative interference as well as positive interference in serum HBsAg(Hepatitis B surface antigen) ELISA(Enzyme-linked immunosorbent assay), but it is unclear that RF causing negative interference is an anomaly produced by a certain ELISA kit or a common property of most of HBsAg ELISA kits.

**Methods:**

Serum models were made by blending HBsAg-positive sera and high- or moderate-concentration RFs sera at the ratio of 1: 9, then one-step and two-step ELISA were adopted to determine HBsAg in serum models.

**Results:**

No matter what kind of kit used, one-step ELISA showed that HBsAg S/CO( sample/cut off) values in serum models were significantly lower than original values. Bivariate correlations tests showed decline rates of HBsAg S/CO Values were not associated to serum RF concentrations ranging from 288 to 3560 IU/mL. HBsAg converted to be negative in 69.80% serum models with original-value ranging from 1.00 to 10.00, and in 2.68% serum models with higher original-value. RF causing decline of HBsAg S/CO value provided by one-step ELISA was more obvious than that provided by two-step ELISA**.**

**Conclusions:**

It is concluded that susceptibility of all HBsAg ELISA assays to interference from RF, leading to predominantly lower and in some cases "false-negative" results, and moreover, the lower the original HBsAg S/CO Value, the higher the false-negative rate.

## Introduction

Rheumatoid factor (RF), a kind of autoantibody against the fragment c portion of IgG, can be of any isotype of immunoglobulins i.e. IgA, IgG, IgM, IgE, IgD[1,2]. Although serum RF levels are elevated in about 2% of healthy people and 20% of people over 60 years old, they are thought to be highly relevant in rheumatoid arthritis. High levels of serum RF occur in about 80% of patients with rheumatoid arthritis[2]. The higher the levels of serum RF, the greater the likelihood of destructive articular disease. It is also found that serum RF levels are elevated in Sjogren's syndrome, systemic lupus erythematosus, systemic sclerosis, dermatomyositis, chronic hepatitis, and primary biliary cirrhosis[2].

RF is sometimes mentioned as an important factor causing positive interference in immunoassay. In two-site immunoassays, RF can bridge the capture antibody and HRP(Horseradish peroxidase)-labeled antibody together and falsely increase the patient’s value[3,4]. Immunoassays using either polyclonal or monoclonal antibody can be affected. In case of RFs, false elevated results arise from the binding of RFs to the fragment c portions of antibodies. The presences of RFs in serum can cause falsely elevated analyte levels in troponin immunoassays [5-7], thyroid function tests [8], tumour marker immunoassays[9,10] and cytokine immunoassays[11,12]. 

The hepatitis B surface antigen (HBsAg) is the first marker that appears in the blood following infection with hepatitis B virus (HBV). The presence of HBsAg in human serum indicates an ongoing HBV infection, either acute or chronic. Testing of additional HBV markers, such as the hepatitis B E antigen, is adopted to define the specific disease state. HBsAg immunoassays are used not only to diagnose HBV infections but also to monitor the course of the disease and the efficacy of antiviral therapy[13,14].

Enzyme-linked immunosorbent assay(ELISA) is widely used to determine the presence of serum HBsAg in China. Investigation from National Center of Clinical laboratory shows 980 out of 1178(83.19%) adopted ELISA to determine serum HBsAg in 2012(www.clinet.com.cn). The top three HBsAg ELISA kits used in clinical laboratory are provided by Kehua Bio-Engineering Co.,Ltd.(Shanghai,China), InTec Xinchuang Science and technology Co.,Ltd.(Xiamen,China) and Wantai Biological Pharmacy Enterprise Co., Ltd.(Beijing, China). Generally RFs are reported to cause positive interference as above in immunoassay[3-12]. Recent study in our study group found that high-concentration RFs led to negative interference as well as postive interference in serum HBsAg ELISA[15], but the negative interferences were only found in serum models with high-concentration RFs by using InTec Xinchuang ELISA kit. It is unclear that RFs causing negative interference is an anomaly produced by InTec Xinchuang ELISA kit or a common denominator of most of serum HBsAg ELISA kits. In this study, we determined whether high-concentration RFs cause negative interference in serum HBsAg ELISA by using six HBsAg ELISA kits purchased from six respective companies, which including the top three companies. In addition, we investigated if moderate-concentration RFs cause negative interference like high-concentration RFs.

## Materials and Methods

### Serum samples

All blood samples were taken at 7 A.M in Union Hospital, Tongji Medical College, Huazhong University of Science and Technology, then incubated at 37°C for half an hour immediately. Sera were isolated with centrifugation for 10 minutes at 4,000rpm/min and stored at -20°C. Eighty Six RF-positive sera (RF≥288 IU/ml), forty five HBsAg-positive sera and twenty normal sera(HBsAg, HBeAg, anti-HBs, anti-HBe, anti-HBc and HBV DNA were all negative) were collected for the subsequent experiments. The RF-positive sera were absorbed with human IgG sensitization latex particles as before[15], and the absorbed sera were determined to be double-negative of HBsAg and anti-HBs by one-step ELISA. 

### Ethics statement

The study was approved by the Ethics Committee of Union Hospital, Tongji Medical College, Huazhong University of Science and Technology. All patients have signed an informed consent.

### Making serum models consisting of HBsAg and moderate- or high-concentration RFs

In this study, 346 serum models were made. Of these, 327 were made as following: blending 25μl of HBsAg-positive sera with 225μl of moderate- or high-concentration RFs sera in a 1.5-ml microcentrifuge tube, then centrifuging for 10 minutes at 4,000rpm/min and collecting supernatant for subsequent experiments. The process in making the other 19 serum models were basically same as above expect serum volume doubled. Control models were made by mixing the same HBsAg-positive sera with normal mixed sera at the ratio of 1:9 respectively.

### Quantification of serum RF levels

Serum RF levels were quantitated using BNⅡsystem and N Latex RF Kit(Siemens Healthcare Diagnostics Inc., Newark, U.S.A.) according to the instruction manual. 

### Determination of serum HBsAg using one-step ELISA

Serum HBsAg was determined with six commercial assay kits listed as following: I (InTec Xinchuang, Xiamen, China); II(Kehua, Shanghai,China); III(3V, Weifang, China);IV(Lizhu, Zhuhai, China); V(Rongsheng, Shanghai,China);VI(Wantai, Beijing, China). One-step ELISAs were employed in all of these kits. The brief operation steps were listed as following: firstly, the kit was equilibrated at room temperature for 30 mins; secondly, certain volume of sample buffer and serum were added into each micropore sequentially(see Table 1), then mixed and incubated for 1 hour at 37°C; thirdly, 50μl HRP(Horseradish peroxidase)-labeled anti–HBs was added and incubated for 30 min at 37°C,then micropores were washed for 5 times with the kit's dedicated buffer before adding 50μl colour developing reagent A and 50μl colour developing reagent B; finally, the reaction was stopped using terminator agent after the incubation at 37°C for 30 min. ELx808 Absorbance Microplate Reader(Biotek Inc., Winooski, U.S.A) was employed determine the integrated optical density. 

**Table 1:** Bascial characteristics of six ELISA kits for HBsAg.

Kits	Method	Sample buffer(μl)	Serum(μl)
I	Two-site ELISA	20	100
II	Two-site ELISA	0	75
III	Two-site ELISA	0	100
IV	Two-site ELISA	0	100
V	Two-site ELISA	0	100
VI	Two-site ELISA	20	100

### Determination of serum HBsAg using Two-step ELISA

In order to disclose mechanism which RFs cause negative interference in serum HBsAg ELISA, two-step ELISA was also employed in this study. Two-step ELISA was basically the same as one-step ELISA except for the additional wash step before adding 50μl of HRP-labled anti–HBs.

### Statistical analysis

Statistical analysis was performed with the SPSS 15.0 software package. Wilcoxon Signed Ranks Test and student T Test were used for comparison of continuous data, whereas Chi-square Test was used to analyze categorical data. In addition, Bivariate Correlations Test was employed to analyze correlation between RF concentrations and decline rate of HBsAg in this study. p≤0.05 was considered to be statistically significant.

## Results

### HBsAg-positive rate in RF-positive sera

A total of 64 serum samples, which RF concentration ranged from 288 to 3560 IU/ml, were determined with six respective ELISA kits. According to instruction manuals, 1.00 was assigned to be the cut-off values to discriminate HBsAg-positive and -negative samples. The highest HBsAg-positive rate(7.81%) was provided by kit I, whereas no one sample was identified to be HBsAg-positive by using kits III, IV, V and VI. S/CO(sample/cut off) values of positive HBsAg were presented in Table 2.

**Table 2:** S/CO values of positive HBsAg in RF-positive sera.fr.

RF levels(IU/mL)	HSCV-I	HSCV-II	HSCV-III	HSCV-IV	HSCV-V	HSCV-VI
1210	2.56	1.63	0.65	0.26	0.31	0.01
2880	2.30	0.02	0.07	0.01	0.21	0.37
2460	1.85	0.01	0.07	0.51	0.37	032
288	3.40	0.20	0.20	0.31	0.22	N
1010	2.74	0.73	0.15	0.26	0.51	0.25
Positive rate	7.81%	1.56%	0.00%	0.00%	0.00%	0.00%

“N” means not test due to insufficient sample.

The highest positive rate of HBsAg from RF is underlined.

HSCV-I, - II, - III, -- IV, - V and - VI mean HBsAg S/CO values provided by ELISA kits I, II, III, IV, V and VI, respectively.

### RF causing negative interference in serum HBsAg ELISA

327 serum models consisting of HBsAg and moderate- or high-concentration RFs were made. Of these,124 included high-concentration RFs(RF>1000IU/ml), the remaining owned moderate-concentration RFs(288<RF<1000IU/ml). One-step ELISA was used to determine HBsAg in these serum models and control models simultaneously. Regardless of kit used in this study, Wilcoxon Signed Ranks Test showed that HBsAg S/CO values of serum models were significantly lower than original values (see Table 3). In addition, 22(6.73%) serum models were found to have higher HBsAg S/CO values than original.

**Table 3:** Median HBsAg S/CO values of serum models provided by six ELISA kits.

Kits	RF levels (IU/mL)	Model number	Median values	Original value	P value	Sum of positive^§^ ranks
I	288-3560	50	17.12	8.89	P=0.00	3
II	288-3560	87	12.74	5.7	P=0.00	3
III	288-3560	69	11.92	1.75	P=0.00	2
IV	583-3560	38	18.05	8.76	P=0.00	5
V	288-2580	37	33.38	13.77	P=0.00	9
VI	288-2580	46	24.18	7.41	P=0.00	0

^§^ Positive means that HBsAg S/CO value in serum model is greater than original value.

Original values are HBsAg S/CO values in control models.

Then we combined into all the serum models to analyze whether RF concentrations correlated with decline rates of HBsAg S/CO values. Bivariate correlations tests showed the decline rates were not associated to serum RF concentrations when serum RF concentrations ranged from 288 to 3560 IU/mL(r=-0.05,p=0.42>0.05). Wilcoxon Signed Ranks Test also showed that there was no significant difference of the decline rates between moderate- and high-concentrations RF groups (0.44 vs. 0.47; Z=0.86,P=0.45>0.05). Sixty serum models, buried in the 327, were made by blending 15 different levels of RF (583<RF<3560IU/ml) and a same context of HBsAg-positive serum, respectively. Bivariate correlations tests also showed serum RF concentrations were also not associated to the decline rates of HBsAg S/CO values provided by kits I(r=0.10, p=0.37>0.05), II(r=0.10,p=0.71>0.05), III(r=0.13,p=0.65>0.05) and IV(r=0.26,p=0.34>0.05).

In this study, HBsAg converted to be negative in 69.80% serum models with original value ranging from 1.00 to 10.00, and in 2.68% serum models with higher original value. HBsAg-negative rates were significantly higher in serum models that original-value ranged from 1.00 to 10.00 than those with higher original value (see Table 4). But there was no obvious difference of HBsAg-negative rates between moderate- and high-concentration RF groups(16.10% vs. 23.20%, χ^2^=2.33,P=0.13>0.05). 

**Table 4:** HBsAg negative rate of serum model in study group.

Original value	HNR-I	HNR-II	HNR-III	HNR-IV	HNR-V	HNR-VI	Total
1-10	O/13	15/30	20/27	0/1	11/11	15/20	61/102*^#^
10-20	O/21	1/41	5/21	0/21	0/3	0/0	6/107^§^
≥20	0/16	0/16	0/21	0/16	0/23	0/26	0/118
Total	0/50	16/87	25/69	0/38	11/37	15/46	67/327

Compared to group "Original value ≥20", *χ^2^=97.64, P=0.00<0.05; ^§^χ^2^=6.79, P=0.01<0.05.

Compared to group "Original value 10-20", ^#^χ^2^=70.42, P=0.00<0.05;

HNR-I, - II, - III, - IV, - V and - VI mean HBsAg negative rates of serum models provided by ELISA kits I, II, III, IV, V and VI, respectively.

### HBsAg S/CO values provided by one-step ELISA decreased more obviously than those provided by two-step ELSA

A total of 19 serum models, which consisted of HBsAg and moderate- or high concentration RFs, were determined with four out of the six kits by using two-step ELSA and one-step ELISA, respectively. Comparing with control models, serum models were presented with lower HBsAg S/CO value irrespective of two-step ELSA and one-step ELISA, but RF caused decline of HBsAg S/CO value provided by one-step ELISA more obviously than by two-step ELSA(see Table 5)

**Table 5:** Comparison of decline rate of HBsAg S/CO values from RF between group “one-step ELISA” and “two-step ELISA”.

Kits	S1	O1	P1	D1	S2	O2	P2	D2	P value
II	2.12	15.44	0.00	0.67	2.23	11.75	0.00	0.60	0.008
III	0.34	5.59	0.002	0.73	2.30	7.76	0.008	0.29	0.003
IV	3.34	14.10	0.00	0.63	6.23	17.72	0.00	0.42	0.00
V	0.65	14.85	0.00	0.78	1.83	9.18	0.00	0.53	0.00

RF levels range from 327 to 8090 IU/ml;

“S1” and “S2” mean median HBsAg S/CO values in serum models provided by one-step ELISA and two-step ELISA; “O1” and “O2” mean median original-values provided by one-step ELISA and two-step ELISA. “P1” and “P2” were calculated by Signed Ranks Test and used to identify whether there is significant difference between “S1” and “O1”, and between “S2” and “O2”, respectively.

“D1” and “D2” was calculated using formula D=(X1- X^2^)/X1, X1 and X^2^ represent original and measurable values of HBsAg S/CO in serum models

P value was calculated by Paired Samples T Test or Signed Ranks Test(in group III) and used to identify whether there is significant difference between “D1” and “D2”.

## Discussion

In this study, HBsAg were determined in 64 serum samples with moderate- and high-concentration RFs using one-step ELISA according to instruction manuals provided by the six ELISA kits, respectively. Positive HBsAg was only provided by kits I and II, and the highest HBsAg-positive rate was 7.81%. Because of insufficient volume, a few samples were not conducted determination of HBsAg simultaneously using six respective ELISA kits. Even so, our results still supported RF was able to cause positive interference in HBsAg ELISA [3,4], and moreover, different ELISA kits had different ability to resist positive interference from moderate- and high-concentration RFs.

Experience in our laboratory showed serum samples with high-concentration RFs caused false decrease of HBsAg S/CO value[15]. In this study, we investigated whether moderate-concentration RFs in serum resulted in negative interference in HBsAg ELISA as well as high-concentration RFs, in addition, we speculated the negative interference was a common denominator of HBsAg ELISA kits. Therefore, we made 327 serum models consisting of moderate- to high-concentration RF and HBsAg, whereas control models were blended these HBsAg-positive sera with normal mixed sera at the same ratio respectively. Comparing to original values, HBsAg S/CO values of serum models decreased significantly regardless of ELISA kit used in this study. In addition, we also observed that HBsAg S/CO values increased in 22(22/327) serum models, which is interpreted as RF can by sheer chance bridge capture antibodies and HRP-labeled anti-HBs antibodies together and falsely elevates HBsAg S/CO values[16]. Therefore, we can conclude that moderate- to high-concentration RFs cause both positive and negative interference in HBsAg ELISA, furthermore, negative interference is much more common than positive interference. Because decline of HBsAg S/CO values was present in HBsAg ELISA regardless of kits used in this study, we put all the serum models together and analyzed whether decline rates of HBsAg S/CO values were associated to serum RF levels. When RF concentrations ranged from 288 to 3560, decline rates of HBsAg S/CO values were not associated to serum RF concentrations significantly. In order to exclude a possible bias introduced by the diversity/variability from HBsAg-positive sera, 60 serum models consisting different levels of RF and a same context of HBsAg-positive serum, were determined with four different HBsAg ELISA kits. Although insufficient volume of RF-positive sera prevents us from determining with the remaining two kits, The results support further that the decline rates of HBsAg S/CO values are not associated to serum RF concentrations(RF>288 IU/ml). Interestingly, when serum models were divided into three groups according to original values, we found that the lower the HBsAg S/CO original-Value, the higher the false-negative rate of HBsAg in serum models. Therefore, it is reasonable to conclude that moderate- and high-concentration RFs cause false HBsAg-negative when original serum HBsAg levels are relative low.

Since it is very common that moderate- and high-concentration RFs cause negative interference in serum HBsAg ELISA, It is reasonable to speculate that binding of RF and capture antibody or HRP labeled anti-HBs can inhibit specific binding between HBsAg and capture antibody or HRP labeled anti-HBs,. In order to disclose which make contribution(or greater contribution) for inhibition and negative interference, two-step ELISA as well as one-step ELISA was conducted to determination of HBsAg. Comparing to one-step ELISA, additional washing step was inserted before adding HRP-labeled anti–HBs in two-step ELISA. Because of additional washing, only RFs binding with capture antibodies were retained in two-step ELISA, so influence of binding of RF and HRP labeled anti-HBs on negative interference disappear. Based on decline rates of HBsAg S/CO values, RF was found to cause decline more significantly in one-step ELISA than in two-step ELISA. We then concluded (i)RF have ability to bind capture antibody as well as HRP-labeled antibody, (ii)the binding of RF and capture antibody makes contribution for inhibiting formation of complex consisting of HBsAg and capture antibody, (iii) the binding between RF and HRP-labeled antibody is also capable of inhibiting on specific interaction between HBsAg and HRP-labeled antibody. Because RF is showed to react against Fab portion of antibody, we speculate that steric hindrance from RF is responsible for inhibition and negative interference in HBsAg ELISA.

This study shows susceptibility of all HBsAg ELISA assays to interference from RF , leading to predominantly (but not exclusively) lower and in some cases "false-negative" results, and moreover, the lower the original HBsAg S/CO Value, the higher the false-negative rate. Care may be needed in interpreting negative HBsAg tests particularly in patients > 60 years of age with rheumatological diseases and in whom serum RF concentration can be high. If moderate- and high-concentration RFs is present, we highly recommend to measure serum HBsAg after absorption with human IgG sensitization latex particles or other blocker. 
